# Fear of falling is as important as multiple previous falls in terms of limiting daily activities: a longitudinal study

**DOI:** 10.1186/s12877-021-02305-8

**Published:** 2021-06-07

**Authors:** Minhui Liu, Tianxue Hou, Yuxiao Li, Xiaocao Sun, Sarah L. Szanton, Lindy Clemson, Patricia M. Davidson

**Affiliations:** 1 Central South University Xiangya School of Nursing, 172 Tongzipo Road of Yuelu District, Hunan 410013 Changsha, China; 2 Johns Hopkins University School of Nursing, MD Baltimore, USA; 3 Johns Hopkins University Bloomberg School of Public Health, MD Baltimore, USA; 4 The University of Sydney, New South Wales Camperdown, Australia

**Keywords:** Fear of falling, Limited daily activities, Older adults, Previous falls

## Abstract

**Background:**

Fear of falling and previous falls are both risk factors that affect daily activities of older adults. However, it remains unclear whether they independently limit daily activities accounting for each other.

**Methods:**

We used the data from Round 1 (Year 1) to Round 5 (Year 5) of the National Health and Aging Trends Study. We included a total of 864 community-dwelling participants who provided data on previous falls, fear of falling and limited activities from Year 1 to Year 5 and had no limited daily activities at Year 1 in this study. Previous falls and fear of falling were ascertained by asking participants how many falls they had in the past year and whether they had worried about falling in the last month. Limited daily activities included any difficulties with mobility (e.g., going outside), self-care (e.g., eating), and household activities (e.g., laundering). Generalized estimation equation models were used to examine whether previous falls and fear of falling independently predicted development of limited daily activities adjusting covariates.

**Results:**

Participants were mainly between 65 and 79 years old (83 %), male (57 %), and non-Hispanic White (79 %). Among participants who had multiple falls in Year 1, 19.1-31 %, 21.4-52.4 %, and 11.9-35.7 % developed limitations in mobility, self-care, and household activities during Year 2 to Year 5, respectively. Among those who had fear of falling in Year 1, 22.5-41.3 %, 30.0-55.0 %, and 18.8-36.3 % developed limitations in mobility, self-care, and household activities during Year 2 to Year 4, respectively. Fear of falling independently predicted limitations in mobility (Incidence rate ratio [IRR]: 1.79, 95 % CI: 1.44, 2.24), self-care (IRR: 1.25, 95 % CI: 1.08, 1.44) and household activities (IRR: 1.39, 95 % CI: 1.08, 1.78) after adjusting for previous falls and covariates. Multiple previous falls independently predicted limitations in mobility (IRR: 1.72, 1.30, 2.27), self-care (IRR: 1.40, 95 % CI: 1.19, 1.66) and household activities (IRR: 1.36, 95 % CI: 1.01, 1.83) after adjusting fear of falling and covariates.

**Conclusions:**

Fear of falling seems to be as important as multiple previous falls in terms of limiting older adults’ daily activities.

## Introduction

About 18 million United States older adults have limited daily activities, and this number is expected to increase rapidly in the coming decades [1, 2]. Limited daily activities arise when older adults have difficulty or need help performing daily activities. Limitations in performing daily activities are common in areas such as mobility, self-care, and household activities. For example, about 18 % of United States older adults have difficulty for mobility or self-care activities, and 25 % need help with household activities [2]. Limited daily activities can have adverse effects on older adults’ health, and it has been a major limitation to social participation and disability [3].

Fear of falling and previous falls are two important and different predictors of limited daily activities in older adults [4–6]. Fear of falling is a persistent concern about falling, which may lead older adults to avoid activities that they are still able to do [7]. About 20–60 % of community-dwelling older adults have fear of falling, and 20–55 % report limited daily activities due to fear of falling [8]. About 30–50 % of independently living older adults fears of falling whether or not they have previous falls [9]. In the short term, fear of falling may seem to reduce the occurrence of falls by avoiding activities, and may protect older adults from danger. For example, after an injury, fear of falling may prevent older adults from engaging in relatively dangerous activities [10, 11]. In the long run, it may limit the ability of older adults to perform daily activities, resulting in physical function decline [12, 13]. Studies also have shown that previous falls have adverse effects on function and loss of confidence, which may lead to limited daily activities [14]. There is a vicious cycle between fear of falling and previous falls in that they predict future falls and make future falls more likely. However, it is still unknown whether previous falls or fear of falling put more risks on limited daily activities.

Although there has been extensive research on fear of falling and previous falls as major risk factors resulting in limited daily activities, it is not clear whether fear of falling independently predicts limited activities controlling for previous falls, and vice versa from longitudinal studies. One longitudinal study found that the effects of fear of falling on declines of activities of daily living (ADLs) in non-fallers were as strong as those in fallers. However, it did not evaluate the effects of falls on declines in ADLs, and a clinical sample from a trial was used [15]. In addition, a cross-sectional study with a sample size of 251 older adults showed that compared with one previous fall, multiple previous falls may have a stronger relationship with limited daily activities [16]. However, this study limited the testing of the causal relationship between previous falls, fear of falling and limited daily activities. This could mean that multiple previous falls are a powerful predictor of limited daily activities than one previous fall. So it is important to identify their independent effects on limited daily activities because fear of falling is a psychological construct, and one or multiple falls are physical [7]. Both are modifiable but may require different types of preventive strategies [17, 18].

Studies have shown that sex is a risk factor for falls and fear of falling [19, 20]. Women are more likely to fall than men, and women are more likely to have fear of falling [19, 20]. Because of sex differences in physiological vulnerability, fear of falling as a psychological construct may differ in the predictive ability of limited daily activities depending on sex. Considering the negative impact of fear of falling on older adults, and the necessity of developing tailored interventions, it is valuable to determine whether fear of falling limits older adults’ daily activities differently by sex.

The purpose of this study is to examine (1) the independent effects of one or multiple previous falls on limited daily activities adjusting for covariates and fear of falling in older adults, and (2) the independent effects of fear of falling on limited daily activities adjusting for covariates and one or multiple previous falls in older adults, and (3) whether the predictive ability of fear of falling in limited daily activities differs by sex.

## Methods

The study was longitudinal, and aimed to examine whether previous falls and fear of falling independently predict limited daily activities among older adults. We used data from Year 1 to Year 5 of the National Health and Aging Trend Study (NHATS), which is a nationally representative survey of United States Medicare Beneficiaries aged 65 or older. The study was designed to investigate differences in the trajectory of various at-risk subgroups and sub-healthy older adults and deepen understanding of the gradual aging of older adults towards the end of life [21]. The data were collected in 2011 (*N* = 8,245) and then tracked annually [22]. Information on participants’ physical and cognitive abilities, daily activities, and physical environment were collected via in-person interviews. The Johns Hopkins University Institutional Review Board approved the research protocol. We included a total of 864 community-dwelling participants who provided data on previous falls, fear of falling and limited activities from Year 1 to Year 5 and had no limited daily activities at Year 1 in this study. Compared to participants who were included in this study, the excluded participants, had less education, and had more chronic diseases, hospitalizations with poorer health status. The excluded participants were also less obese, engaged in less vigorous activity.

### Measures

#### Dependent variables

Limited daily activities included any difficulties or needing help with mobility (going outside, getting around inside, and getting in and out of bed), self-care activities (eating, bathing, toileting, and dressing), household activities (laundering, shopping, preparing meals, managing bills, and taking medications). For mobility and self-care activities, participants were asked the extent to which activities were accomplished independently in the last month, the frequency of using assistive devices to accomplish tasks last month, how often they got help from others, how much difficulty it was to independently accomplish the task, and compared to a year ago whether they accomplished the task more often, less often, or about the same. For household activities, participants were asked if anyone else had accomplished the activities with them in the last month for some reasons such as health, how difficult to accomplish the activities by themselves, and whether they accomplished activities by themselves more or less often than a year ago [2].

We classified limitations in mobility, self-care, and household activities into four levels: (1) No limitations on daily activities, which mean no use of equipment, no difficulties in activities, no need for help, or no reduction in frequency. (2) Need to use the equipment without encountering difficulties or receiving help from others. (3) Have difficulty working alone, but does not accept help from others. (4) Need help from others due to health, function, or living in nursing home [2]. Numbers of activity limitations in mobility, self-care, and household activities Participants were further classified into two groups based on these four levels: (1) No limitation in daily activities (level 1). (2) Have limitations in daily activities (level 2, 3, and 4). With these criteria, we also created variables to indicate the mean numbers of activity limitations in mobility, self-care, and household activities, respectively.

#### Independent variables

Previous falls were measured with the questions: “In the last 12 months, have you (or sample person, in the case of the surrogate interview) fallen down?” The answer was yes or no. “In the last 12 months, have you (or sample person, in the case of the surrogate interview) fallen down more than one time?” The answer was yes or no. A fall was defined as having a fall, trip, or slip on the floor, or lower level because of losing balance.

Fear of falling was measured by asking participants to the question: “In the last month, did you (or sample person, in the case of the surrogate interview) worry about falling down?” The responses were yes or no and we classified it as a binary variable.

#### Covariates

##### Sociodemographics

Information on age group, sex, race/ethnicity, education and living arrangements were collected.

##### Health-related factors

The BMI was calculated by dividing the current body weight in kilograms by the square of the height in meters, and obesity is defined as a BMI greater than or equal to 30 kg/m^2^. Chronic diseases were assessed by asking participants whether doctors had diagnosed any of the following: heart attack, heart disease, high blood pressure, arthritis, osteoporosis, diabetes, lung disease, stroke, cancer and dementia/Alzheimer’s disease. Hospitalizations were evaluated by asking participants if they had been hospitalized in the past year. Cognitive function was divided into three categories: no dementia, possible dementia, and probable dementia, according to the NHATS dementia classification scheme. Depression was assessed by the Patient Health Questionnaire (PHQ-2) scale.

##### Behavioral factors

Smoking status was determined by the response to ever smoked or currently smoke cigarettes regularly, at least 1 cigarette a day. Vigorous activities were assessed by asking participants whether they ever spend time on vigorous activities that increased your heart rate and made you breathe harder in the last month (e.g., working out, swimming, running or biking, or playing a sport).

### Data analysis

We first used frequencies and proportions to describe the demographic and health-related characteristics of all participants and then use Chi-square tests to compare these characteristics between those with one or multiple previous falls or without previous falls, and between those with and without fear of falling reported in Year 1. We then calculated the percentages of participants who developed limitations in mobility, self-care, and household activities at follow-ups by one or multiple previous falls and fear of falling status. We also used Chi-square tests to examine the associations between one or multiple previous falls and fear of falling in Year 1 and development of activity limitations in mobility, self-care, and household activities at follow-ups.

To investigate the independent effects of previous falls and fear of falling on development of limited daily activities, we used generalized estimating equations (GEEs) with a Poisson distribution to model the prior-wave (period *t*-1) falls or fear of falling on the probability of having limited daily activities at each current wave (period *t*) assessment using the log link function. GEE models are an extension of generalized linear models for analyzing longitudinal data accounting for the correlation of repeated measures. Using the lagged independent variables allow us to determine the temporal effects of falls and fear of falling on development of limited daily activities in this study. Independent effects of one or multiple previous falls and fear of falling were examined in three steps. First, we included one or multiple previous falls as the main predictor of each limited daily activity in the models, only adjusting for covariates (Model 1). Second, we examined fear of falling as the main predictor of limited daily activity in the model, only adjusting for covariates (Model 2). Finally, we included both one or multiple previous falls and fear of falling as predictors of each limited daily activity adjusting for covariates to examine their independent effects on limited daily activities (Model 3).

To examine whether the predictive ability of fear of falling in limited daily activities is different by sex, we also used GEEs with a Poisson distribution. First, we examined whether fear of falling and previous falls predicted limited daily activities stratified by sex as a binary variable (namely strategy analysis). Then, we tested the significance of the interaction term between fear of falling and sex, and the interaction term between previous falls and sex using models described above (namely analysis with interaction terms).

Since missing values on covariate variables were less than 1.6 %, we did not apply any techniques to handle them. All analyses were performed using Stata version 14.0 (Stata Corp, College Station, Texas, USA); *p* < .05 (two-tailed) was used to indicate statistical significance. Incidence rate ratios (IRRs) and 95 % confidence intervals (CIs) were reported from GEE models.

## Results

### Sample characteristics

The participants without limited daily activities in Year 1 were mostly aged between 65 and 79 years old. About 57 % were male, 60 % received some college education or higher, the majority were non-Hispanic White (79 %), and about 26 % lived alone. They were generally healthy, with only about 23 % being obese, 8 % ever smoked, 18 % having none chronic condition, 88 % not hospitalized in last year, and about half performing vigorous activities in the last year. About 18 and 9 % of participants had previous falls and fear of falling in Year 1, respectively. Participants who fell last year were more likely to be female and have a smoking history. Those who had fear of falling tended to be older females and with 3 or more chronic conditions (Table 1).

**Table 1:** Sample characteristics by previous falls and fear of falling status in Year 1 (n, %)

Variables	All	Previous Falls in Year 1				Fear of Falling in Year 1		
		No	One fall	Multiple falls	***P*** value	No	Yes	***P*** value
Age (y)					0.238			**0.039**
65-79	719 (83.2)	588 (81.8)	96 (13.4)	35 (4.9)		659 (91.7)	60 (8.3)	
80-90+	145 (16.8)	126 (86.9)	12 (8.3)	7 (4.8)		125 (86.2)	20 (13.8)	
Sex					0.106			**0.012**
Female	371 (42.9)	295 (79.5)	54 (14.6)	22 (5.9)		326 (87.9)	45 (12.1)	
Male	493 (57.1)	419 (85.0)	54 (11.0)	20 (4.1)		458 (92.9)	35 (7.1)	
Education					0.104			0.180
Less than high school	133 (15.5)	106 (79.7)	20 (15.0)	7 (5.3)		125 (94.0)	8 (6.0)	
High school graduates	209 (24.3)	173 (82.8)	32 (15.3)	4 (1.91)		184 (88.0)	25 (12.0)	
Some college or vocational school	195 (22.7)	169 (86.7)	16 (8.2)	10 (5.1)		174 (89.2)	21 (10.8)	
Bachelor or higher	322 (37.5)	262 (81.4)	40 (12.4)	20 (6.2)		297 (92.2)	25 (7.8)	
Race					0.079			0.083
White, non-Hispanic	683 (79.1)	562 (82.3)	84 (12.3)	37 (5.4)		618 (90.5)	65 (9.5)	
Black, non-Hispanic	127 (14.7)	111 (87.4)	14 (11.0)	2 (1.6)		121 (95.3)	6 (4.7)	
Hispanic	30 (3.5)	23 (76.7)	7 (23.3)	0		25 (83.3)	5 (16.7)	
Indian/Asian/Native/Hawaii/Other	24 (2.8)	18 (75.0)	3 (12.5)	3 (12.5)		20 (83.3)	4 (16.7)	
Living					0.435			0.317
Alone	220 (25.6)	182 (82.7)	29 (13.2)	9 (4.1)		197 (89.6)	23 (10.5)	
With spouse/partner only	482 (56.1)	394 (81.7)	62 (12.9)	26 (5.4)		442 (91.7)	40 (8.3)	
With others only	77 (9.0)	61 (79.2)	10 (13.0)	6 (7.8)		66 (85.7)	11 (14.3)	
With spouse/partner and with others	81 (9.4)	73 (90.1)	7 (8.6)	1 (1.2)		75 (92.6)	6 (7.4)	
BMI					0.619			0.123
Normal	660 (77.5)	542 (82.1)	85 (12.9)	33 (5.0)		605 (91.7)	55 (8.3)	
Obesity	192 (22.5)	163 (84.9)	22 (11.5)	7 (3.7)		169 (88.0)	23 (12.0)	
Smoking					**0.046**			0.572
No	796 (92.1)	664 (83.2)	93 (11.7)	39 (4.9)		721 (90.6)	75 (9.4)	
Yes	68 (7.9)	50 (73.5)	15 (22.1)	3 (4.4)		63 (92.7)	5 (7.4)	
Vigorous activities					0.643			0.179
No	392 (45.4)	321 (81.9)	49 (12.5)	22 (5.6)		350 (89.3)	42 (10.7)	
Yes	472 (54.6)	393 (83.3)	59 (12.5)	20 (4.2)		434 (92.0)	38 (8.1)	
Number of chronic illnesses					0.334			**0.043**
No disease	157 (18.2)	135 (86.0)	14 (8.9)	8 (5.1)		149 (94.9)	8 (5.1)	
1~3	601 (69.6)	493 (82.0)	82 (13.6)	26 (4.3)		544 (90.5)	57 (9.5)	
≥ 4	106 (12.3)	86 (81.1)	12 (11.3)	8 (7.6)		91 (85.9)	15 (14.2)	
Hospitalization					0.213			0.872
No hospitalization	762 (88.2)	636 (83.5)	91 (11.9)	35 (4.6)		691 (90.7)	71 (9.3)	
At least one hospitalization	102 (11.8)	78 (76.5)	17 (16.7)	7 (6.9)		93 (91.2)	9 (8.8)	
Depression					**0.006**			0.292
No depression	809 (93.9)	676 (83.6)	93 (11.5)	40 (4.9)		737 (91.1)	72 (8.9)	
Depression	53 (6.2)	37 (69.8)	14 (26.4)	2 (3.8)		46 (86.8)	7 (13.2)	
Cognitive function					0.877			0.339
No dementia	789 (91.3)	650 (82.4)	99 (12.6)	40 (5.1)		718 (91)	71 (9)	
Possible dementia	60 (6.9)	51 (85)	7 (11.7)	2 (3.33)		54 (90)	6 (10)	
Probable dementia	15 (1.7)	13 (86.7)	2 (13.3)	0		12 (80)	3 (20)	

### Association of Year 1 previous falls, Year 1 fear of falling (fear of falling), and development of limited daily activities from Year 2 to Year 5

**Fig 1.**
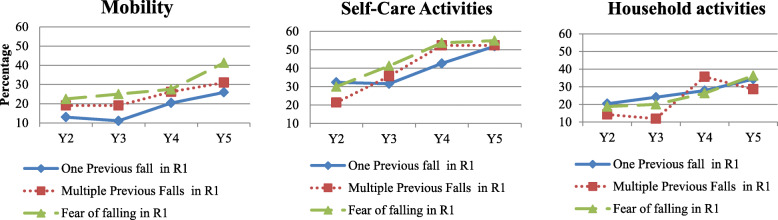
Percentages of participants who developed activity limitations from Year 2 to Year 5

Figure 1 shows the percentage of participants who developed limited daily activities from Year 2 to Year 5 by previous falls and fear of falling status at Year 1. Overall, among participants with reported one or multiple previous falls or fear of falling at Year 1, the percentages of developing limitations in mobility, self-care, and household activities steadily increased from Year 2 to Year 5. For example, among participants who reported a fall at Year 1, 11.1-25.9 % developed limitations in mobility, 31.5-51.9 % experienced limitations in self-care activities, and 20.4-34.3 % had household limited daily activities during follow-ups Year 2 to Year 5. Year 1 multiple previous falls were only significantly associated with a higher percentage of participants who had limitations in self-care activities in Year 4. However, Year 1 fear of falling significantly contributed to developing limitations in mobility, self-care, and household activities in the following years. For example, participants who had fear of falling in Year 1 were more likely to have limitations in mobility since Year 2 and limitations in self-care activities since Year 3 (Fig. 1).

### Independent effects of previous falls and fear of falling on limited daily activities

Table 2 summarizes incidence rate ratio estimates of limited daily activities adjusting for covariates from GEE models. Model 1 and Model 2 showed that both multiple previous falls and fear of falling in the prior year independently predicted development of limitations in mobility, self-care, and household activities a year later after adjusting for covariates. For example, the incidence rate of mobility limitations was 1.96 times greater among those who had multiple times last year compared to those without previous falls (IRR: 1.96, 95 % CI: 1.49, 2.59). Participants who had fear of falling in the prior year also had a significant higher incidence of mobility limitations compared to those without fear of falling (IRR: 1.93, 95 % CI: 1.54, 2.40). After including both previous falls and fear of falling in the adjusted models, multiple previous falls (IRR: 1.72, 1.30, 2.27) and fear of falling (IRR: 1.79, 95 % CI: 1.44, 2.44) still independently predicted subsequent mobility limitations; which was also true for limitations in self-care activities (IRR: 1.40, 95 % CI: 1.19, 1.66) vs. (IRR: 1.25, 95 % CI: 1.08, 1.44), and household activities (IRR: 1.36, 95 % CI: 1.01, 1.83) vs. (IRR: 1.39, 95 % CI: 1.08, 1.78).

**Table 2:** Independent effects of previous falls and fear of falling on limited daily activities

Variables	Model 1	Model 2	Model 3
	IRR [95%CI]	IRR [95%CI]	IRR [95%CI]
Mobility			
One Fall	1.14 [0.91,1.44]		1.09 [0.87,1.36]
Multiple Falls	1.96 [1.49,2.59]***		1.72 [1.30,2.27]***
Fear of Falling		1.93 [1.54,2.40]***	1.79 [1.44,2.24]***
Self-care activities			
One Fall	0.97 [0.85,1.10]		0.95 [0.84,1.08]
Multiple Falls	1.47 [1.25,1.74]***		1.40 [1.19,1.66]***
Fear of Falling		1.31 [1.14,1.51]***	1.25 [1.08,1.44]***
Household activities			
One Fall	1.17 [0.94,1.46]		1.13 [0.91,1.41]
Multiple Falls	1.48 [1.11,1.98]**		1.36 [1.01,1.83]*
Fear of Falling		1.46 [1.14,1.87]**	1.39 [1.08,1.78]*

* *IRR* incidence rate ratio; * *p* < .05, ** *p* < .01, *** *p* < .001

All models adjusted for demographic covariates (age, sex, education, race/ethnicity, living arrangement), health-related covariates (BMI, number of chronic illnesses, hospitalizations, depression, cognitive function), and behavioral covariates (smoking and vigorous activities)

### Predicting ability of fear of falling in developing activity limitations differed by sex

Table 3 summarizes incidence rate ratio estimates of limited daily activities by sex adjusting for covariates from GEE models. The stratified analysis showed that fear of falling in the prior year independently predicted development of limitations in mobility (IRR: 2.07, 95 % CI: 1.58, 2.71), self-care (IRR: 1.50, 95 % CI: 1.24, 1.81), and household activities (IRR: 2.10, 95 % CI: 1.60, 2.76) a year later, and multiple previous falls independently predicted development of limitations in mobility (IRR: 1.67, 95 % CI: 1.14, 2.45) and self-care activities (IRR: 1.49, 95 % CI: 1.22, 1.82) a year later among men after adjusting for covariates. Multiple previous falls in the prior year independently predicted development of limitations in mobility (IRR: 1.83, 95 % CI: 1.23, 2.74), and household activities (IRR: 1.56, 95 % CI: 1.05, 2.32) a year later among women after adjusting for covariates. The analysis with interaction terms showed the similar results, that fear of falling play a more severe role as a predictor on self-care, and household activities in men then in women.

**Table 3:** Independent and interaction effects of previous falls and fear of falling on developing activity limitations by sex

Variables	Stratified Analysis IRR [95%CI]		Analysis with Interaction Term IRR [95%CI]
	Male	Female	
Mobility			
One Fall	1.24 [0.92,1.67]	0.88 [0.62,1.25]	1.09 [0.87,1.37]
Multiple Falls	1.67 [1.14,2.45]**	1.83 [1.23,2.74]**	1.72 [1.30,2.28]***
Fear of Falling	2.07 [1.58,2.71]***	1.59 [1.10,2.30]*	1.53 [1.07,2.18]*
Fear of Falling*Sex			1.33 [0.86,2.04]
Self-care activities			
One Fall	0.95 [0.79,1.13]	0.96 [0.80,1.16]	0.96 [0.84,1.09]
Multiple Falls	1.49 [1.22,1.82]***	1.27 [0.96,1.68]	1.40 [1.19,1.66]***
Fear of Falling	1.50 [1.24,1.81]***	1.05 [0.87,1.28]	1.01 [0.84,1.22]
Fear of Falling*Sex			1.50 [1.16,1.94]**
Household activities			
One Fall	1.32 [0.98,1.77]	1.00 [0.74,1.36]	1.15 [0.93,1.42]
Multiple Falls	1.29 [0.84,1.98]	1.56 [1.05,2.32]*	1.39 [1.03,1.86]*
Fear of Falling	2.10 [1.60,2.76]***	0.98 [0.67,1.44]	0.90 [0.62,1.32]
Fear of Falling*Sex			2.36 [1.51,3.71]***

* *IRR* incidence rate ratio; * *p* < .05, ** *p* < .01, *** *p* < .001

Stratified analysis: demographic covariates (age, education, race/ethnicity, living arrangement), health-related covariates (BMI, number of chronic illnesses, hospitalizations, depression, cognitive function), and behavioral covariates (smoking and vigorous activities)

Analysis with interaction terms: demographic covariates (age, sex education, race/ethnicity, living arrangement), health-related covariates (BMI, number of chronic illnesses, hospitalizations, depression, cognitive function), and behavioral covariates (smoking and vigorous activities)

## Discussion

Using a nationally representative sample of community-dwelling older adults, this study examined the independent effects of fear of falling on development of limited daily activities adjusting for covariates and previous falls, and vice versa. The prevalence rates of previous falls and fear of falling in Year 1 of this study were 18 and 9 %, respectively. These rates were lower than those of previous studies in which fall rates ranged from 29 to 45 % and fear of falling rates ranged from 20 to 60 % [16, 23–27]. This is expected as we excluded participants with limited daily activities in Year 1 for the study purpose.

Our study found that multiple previous falls and fear of falling have the same predictive ability for limited daily activities, but one previous fall was not. Study has shown that the association between fear of falling and activity avoidance is affected by the number of falls in the past year. But older adults who had not experienced a fall also showed limited daily activities. This suggested that some significant portion of fear of falling was inappropriate [16]. Excessive fear of falling can have a major impact on physical performance and lead to poor balance [28, 29]. Delbaere et al. showed that previous falls do not directly lead to changes in daily behaviors, but effective-cognitive variables associated with falls, such as a concern about falling during activity, can mediate this process [30]. Further their modelling also supported that catastrophic fear of falling, such as fear of hip fracture or institutionalization, can lead to activity and mobility restriction without previous falls. Thus, fear of falling as an affective-cognitive variable, whether a worry about falls or a fear of falling, is an important influencing factor leading to changes in daily activities. In some older adults with impaired mobility, fear of falling may result in ADLs impairments, depression, and disability [31–34]. On this basis, our research adds to previous research findings, recognizing that fear of falling can be as important as multiple previous falls in limited daily activities of older adults. Studies have shown that fear of falling can be alleviated by improving cognitive behavior and daily activities in older adults [35, 36]. For older adults who have fear of falling, it might act as barrier for up-taking any fall prevention study or intervention, so lifestyle changes are recommended to achieve effective intervention [37]. At the same time, we should also pay attention to older adults with multiple previous falls to improve their physical fitness and reduce the incidence of falls.

In our study, fear of falling is more predictive of limited daily activities among men than women. In the previous literature, sex is a risk factor of fear of falling, but most of the studies are cross-sectional studies [20, 38]. Friedman and colleagues found that factors such as women, older age, and self-reported previous fall within the last year, are related to the development of fear of falling [27]. Our research results were different from previous studies, which may be because our sample size is mostly male after excluding participants who had limited daily activities in Year 1 .

One strength of this study is that we used a nationally representative longitudinal dataset that allows us to examine the temporal effects of previous falls and fear of falling on the development of limited daily activities. A comprehensive list of covariates were assessed and adjusted for in this study and this allows to provide relatively robust findings. However, there are also some limitations to this study. First, fear of falling was measured by a single item asking if they were worried about falling for which the reliability and validity are still unknown. However, this measure has been widely used in cohort studies and showed consistent findings on health outcomes. Second, recall bias might occur when asking participants about fall experience. We used previous falls as a binary variable instead of a continuous variable, which may lessen the threat to the robustness of our findings. Third, our sample is relatively small after excluding those who missed for follow-ups and had limited daily activities in Year 1, therefore, our study results mainly apply to predominantly healthy and physically active older adults. Yet, fear of falling could have a less relevant influence on daily activities in subjects presenting with a higher number and severity of comorbidities, which may have a greater impact than fear of falling on individuals’ autonomy.

## Conclusions

In summary, while both previous falls and fear of falling are important predictors of limitations in performing daily activities, multiple previous falls and fear of falling make equivalent adverse impact on developing limitations in major daily activities. While paying attention to the fall experience of older adults, we should develop and apply useful strategies to reduce fear of falling among older adults in order to improve their ability to perform daily activities and stay physically independent, and prevent falls.

## Data Availability

The NHATS data analyzed in the current study are available for research purposes at www.nhats.org.

## Abbreviations

ADLs: Activities of Daily Living
NHATS: National Health and Aging Trends Study
GEE: Generalized Estimating Equations
IRR: Incidence rate ratio
BMI: Body Mass Index
CI: Confidence Interval
